# The Macromolecular Machines that Duplicate the *Escherichia coli* Chromosome as Targets for Drug Discovery

**DOI:** 10.3390/antibiotics7010023

**Published:** 2018-03-14

**Authors:** Jon M. Kaguni

**Affiliations:** Department of Biochemistry and Molecular Biology, Michigan State University, East Lansing, MI 48824-1319, USA; kaguni@msu.edu; Tel.: +1-517-353-6721; Fax: +1-517-353-9334

**Keywords:** DNA replication, *Escherichia coli*, inhibitors, replisome, replication fork, initiation, replication origin, DnaA, DnaB, DnaC, primase, DnaG, SSB, DNA gyrase, topoisomerase IV, DNA polymerase I, DNA polymerase III holoenzyme, sliding clamp, clamp loader, DNA ligase

## Abstract

DNA replication is an essential process. Although the fundamental strategies to duplicate chromosomes are similar in all free-living organisms, the enzymes of the three domains of life that perform similar functions in DNA replication differ in amino acid sequence and their three-dimensional structures. Moreover, the respective proteins generally utilize different enzymatic mechanisms. Hence, the replication proteins that are highly conserved among bacterial species are attractive targets to develop novel antibiotics as the compounds are unlikely to demonstrate off-target effects. For those proteins that differ among bacteria, compounds that are species-specific may be found. *Escherichia coli* has been developed as a model system to study DNA replication, serving as a benchmark for comparison. This review summarizes the functions of individual *E. coli* proteins, and the compounds that inhibit them.

## 1. History, and the Current State of Antibiotics in Medicine and the Food Industry

Antibiotics have been critical in the treatment of human disease. Its impact on human health stems from the discovery in 1928 by Dr. Alexander Fleming at St. Mary’s Hospital in London of penicillin that it is effective against a broad range of bacteria but has low toxicity in humans. Penicillin is produced by a specific species of mold named *Penicillium chrysogenum* and is believed to provide a growth advantage to the organism when competing in nature with bacteria for nutrients. Hence, prior to the development and use of antibiotics to treat disease, some bacteria have naturally evolved that are resistant to penicillin. Subsequent work by many laboratories has established that penicillin and its derivatives inhibit the synthesis of the bacterial cell wall. Also developed during the era of penicillin discovery were toxic arsenic derivatives along with sulfonamides such as Prontosil by Bayer Laboratories (Leverkusen, Germany), which is effective against Gram-positive bacteria. Almost a century later, the current family of antibiotics can be separated by their general chemical structures into macrolides, amoxicillins, cephalosporins, fluoroquinones and carbapenems. These compounds inhibit bacterial growth by different mechanisms. They can also be classified by whether they selectively inhibit Gram-negative or Gram-positive bacteria, or work against a wide spectrum of bacterial species, or by their effects on growth (bacteriostatic or bacteriocidal).

It cannot be ignored that improved personal hygiene has dramatically reduced the frequency of bacterial infection in humans. Despite progress in hygiene, acute otitis media (infection of the middle ear) remains the most frequently diagnosed bacterial illness in children with over 20 million physician appointments per year in the United States [1]. The cost of treatment and time lost from school or work due to acute otitis media was almost $3 billion in 1995 and 2006 [2]. In one study, over three-fourths of U.S. children suffered from at least one bout by the third year of age, and nearly half of this group experienced three episodes [3]. The most common bacterial pathogens associated with this illness are *Streptococcus pneumoniae* (40–50%), nonencapsulated *Haemophilus influenzae* (15–30%), and *Moraxella catarrhalis* (3–20%) (reviewed in [4]). Because this illness is recurrent, and is not likely to resolve without therapy [5], a major concern is the complication that arises from persistent infection by antibiotic-resistant bacteria. Viewed more broadly, antibiotic resistance is recognized worldwide as a major threat to human health.

The improper use of modern antibiotics in the treatment of human disease, and their widespread application in agriculture has led to the emergence of antibiotic-resistant bacteria, so called superbugs. In the agricultural industry, antibiotics are used not only to treat bacterial infections in animals but also until recently were added at suboptimal dosages in animal feed and water to promote growth. During the past half century of this practice, daily exposure of livestock to antibiotics and the improper use of antibiotics in human medicine have allowed the selection of multidrug-resistant bacteria. Moreover, the antibiotic-resistant genes are highly mobile, transferring horizontally to other bacteria including pathogens. In the livestock industry, the concern of overuse and inappropriate use of antibiotics has led to the U.S. Food and Drug Administration to establish guidelines to phase out by January 2017 the use of antibiotics in animal feed. The European Union has banned antibiotics since 2006.

In parallel, the World Health Organization in January 2017, formulated for the first time a list of pathogens that pose the greatest threat to human health. The list is divided into three categories by the urgency of need for new antibiotics. In the top tier are species that are multidrug resistant, and include *Acetinobacteria baumannii* (Gram-negative), *Pseudomonas aeroginosa* (Gram-negative), and *Enterobacteriaceae* (large family of Gram-negative pathogens). These bacteria cause bloodstream infections and pneumonia. Species in the second tier cause food poisoning or sexually transmitted disease, and are *Enterococcus faecium* (Gram-negative), *Staphlyococcus aureus* (Gram-positive), *Helicobacter pylori* (Gram-negative), *Campylobacter* spp. (family of Gram-negative pathogens)*,* and *Neisseria gonorrhoeae* (Gram-negative). The third tier includes *Streptococcus pneumoniae* (Gram-positive), *Haemophilus influenzae* (Gram-negative), and *Shigella* spp. (large family of Gram-negative pathogens)

The danger of multidrug-resistant bacteria was recognized by the health care industry well before the declaration by the World Health Organization of the tiered list of pathogens. To attempt to discover novel antibiotics to solve this problem, the traditional method had been cell-based screens, which are labor-intensive. In the 1990s, the pharmaceutical companies took advantage of bioinformatics tools to search bacterial genomes for new drug targets. Together with high-throughput screening assays that measured bacterial growth, the idea was to exploit comparative genomics of bacterial species to identify highly conserved genes that are likely to be essential, followed by predictions of protein function based on the activity of homologous proteins of *E. coli* and *B. subtilis*. The study of these model organisms for many years has led to the identification of genes that are essential for viability. The advantage of this approach is that it is theoretically able to identify all targets of essential pathways. Its weakness is its inability to detect inhibitory compounds that are unable to enter cells or are exported. To date, the comparative genomics approach has had limited success. An alternative to discover novel compounds is to target individual proteins judged to be indispensable for viability using in vitro assays in high throughput screens. These are described in more detail below.

Unfortunately, the major pharmaceutical companies have largely abandoned efforts to develop novel antibiotics because of the low return on investment, the scientific challenges, and clinical/regulatory hurdles [6,7,8,9]. Following the rule in which the primary goal of a company is to increase the wealth of its shareholders, other areas of pharmaceutical research are far more attractive. As examples, patients with diabetes (9.3% of the US population), or high blood pressure (46% of the US population according to the newest guidelines released by the American College of Cardiology/American Heart Association Task Force) receive drug treatment administered over a long period. Hence, drug development for these diseases is justified financially, and it also strives to improve human health. In contrast, treatments for microbial infections are typically much shorter. The predictable income from constant sales associated with long-term treatment of chronic conditions is not the case for medications used to treat microbial infection. The smaller market and smaller volume of sales of antibiotics are explanations cited to justify against antibiotic research for the large-cap pharmaceutical companies. This trend may be changing on the basis of a report released at the 2018 World Economic Forum in which GlaxoSmithKline and Johnson & Johnson are judged to lead in research and development against antimicrobial resistance [10].

## 2. Experimental Approaches to Identify New Antibacterial Compounds

Of interest, some smaller-cap companies have made efforts into the newest generation of antibiotics. These belong to the group of antibacterial drugs mentioned above (macrolides, amoxicillins, cephalosporins, fluoroquinones, and carbapenems). The strategy has been to employ medicinal chemistry to modify existing antibiotics to improve their effectiveness. An alternative approach is random high-throughput screening of chemical libraries that are commercially available and developed specifically for the purpose of drug discovery [6,8]. On finding promising compounds using screens against whole cells or specific target proteins that are known or thought to be essential for viability, their systematic chemical modification may improve their efficacy. Factors to consider in a chemical library are molecular diversity to favor the discovery of an inhibitor, and the drug-like characteristics of compounds (H-bond donors, H-bond acceptors, aromatic center, salt bridge formation, and pharmacokinetics (adsorption, efflux, metabolism, excretion). Taking this approach, GlaxoSmithKline among the larger pharmaceutical companies discovered a novel compound named GSK 299423 that inhibits *Staphylococcus aureus* DNA gyrase and its homologue in other bacteria by a mechanism that is distinct from fluoroquinolone inhibition [11].

Computational methods have also been used that rely on the X-ray crystallographic or NMR structure of a target protein. In the case of DNA polymerase III holoenzyme, in silico docking of a compound and its derivatives onto the hydrophobic pocket of a subassembly of the DNA polymerase named the sliding clamp has led to the identification of several inhibitory compounds. These compounds interfere with the interaction of proteins that interact with the sliding clamp in their recruitment onto DNA. These compounds are described in more detail below.

The approaches of high-throughput screening or molecular docking rely on interrogating a target protein. Such targets are chosen based on our knowledge of a protein’s known role in an essential cellular process. Alternatively, for a bacterial pathogen that is poorly understood at the molecular level, a general strategy has been to compare the DNA sequence of its genome to other bacteria to identify conserved genes. The conservation of a gene suggests that it has been preserved because it is essential for viability. On this issue, 303 genes of *E. coli* have been identified that are essential on the basis that each cannot be genetically deleted without causing lethality [12]. In related work, investigators set out to create a bacterium with the smallest genome. Using DNA from *Mycoplasma mycoides*, a synthetically constructed bacterium that carries only 473 genes in its chromosome was shown to be viable under conditions in which required nutrients were provided in the growth media [13]. Gene editing demonstrated that 375 genes are apparently essential. Their known or speculated functions suggest that at least twenty-seven independent pathways are needed to maintain viability [14]. Hence, each of the 375 genes is a potential target for antibiotic development. By comparison, the families of antibiotics described above inhibit only three essential cellular pathways. Amoxicillins, cephalosporins and carbapenems inhibit synthesis of the bacterial cell wall, macrolides inhibit protein synthesis, and fluoroquinones inhibit DNA gyrase and topoisomerase IV. 

Compared with the experimental strategies summarized above, a novel method of iChip technology of culturing bacteria in soil led to the discovery of teixobactin that inhibits cell wall synthesis by binding to lipid II and III that are cell wall precursors [15]. Perhaps this and other modern technologies may uncover new molecules that inhibit essential biochemical pathways that have not been exploited. It is unfortunate that teixobactin is not effective against Gram-negative bacteria. Many species on the 2017 World Health Organization priority list of bacterial pathogens are Gram-negative.

## 3. DNA Replication Proteins, of Which Many Are Members of the AAA+ Family of ATPases, as Targets for Drug Discovery

The experimental approaches described above target either whole cells or specific proteins using biochemical assays. The problem with whole cell screens is that it conflates the issue with potential problems of adsorption, efflux, metabolism, and excretion of a compound. These individual issues may be separately addressed by modifying the compound to improve its effectiveness. If the target protein interacts directly with other proteins that function in a biochemical pathway, and undergoes conformational changes induced by these interactions, the focus on a specific protein will not identify compounds that block these processes. Indeed, only a very limited number of studies have exploited the proteins that act in concert in a vital biochemical pathway. Considering that DNA replication is essential for the viability of all organisms, and requires several macromolecular machines acting at different stages [16,17,18,19], this biochemical process is an attractive system for investigation. In support, a number of antibiotics that are described in more detail below act on specific proteins that are needed for duplication of the *E. coli* chromosome. The focus on these proteins draws on the wealth of knowledge about them from studies of *E. coli* as a model system. As a brief introduction to these replication proteins, they are DnaA, DnaB, DnaC, primase (DnaG), single-stranded DNA binding protein (SSB), DNA polymerase III holoenzyme, DNA gyrase, DNA polymerase I, and DNA ligase. Except for SSB, DNA polymerase I and DNA ligase, the remainder are ATP binding proteins, but DnaA, DnaC, and the δ and δ’ subunits of DNA polymerase III contain distinguishing amino acid motifs of the AAA+ family of ATPases. The functions of these *E. coli* proteins in DNA replication and compounds that specifically inhibit individual proteins, including inhibitors of DNA polymerases of Gram-positive bacteria, are described below and summarized in Table 1. Some compounds are specific inhibitors of the proteins of *E. coli* and Gram-negative bacteria, whereas others show a broader range of inhibition. Readers are also directed to recent reviews on antimicrobial compounds and those in development against pathogenic bacteria [20,21,22,23,24,25,26].

## 4. SSB

SSB is essential for DNA replication, binding specifically to single-stranded DNA after the parental duplex DNA has been unwound [55]. In addition to protecting the single-stranded DNA from nucleases as it is being copied, SSB also plays a role in DNA recombination and DNA repair and inhibits the formation of aberrant DNA structures. The active form of SSB is a homotetramer. In its functions, SSB interacts with a variety of proteins via a domain near its C-terminus [56,57]. As examples, SSB interacts directly with the α and χ subunits of DNA polymerase III holoenzyme [58,59,60,61,62,63,64,65,66], and with other DNA polymerases of *E. coli* (DNA polymerases II, IV and V) [67,68,69], proteins (PriA, PriB, and PriC) that function in restarting collapsed replication forks [27,58,64,65,70,71,72], and primase [61,73]. The interaction of SSB with primase is critical for primase to remain bound to the primer it has synthesized, which involves the association of primase with DnaB [74,75,76,77,78,79,80,81,82,83]. In contrast, the interaction of SSB with the χ subunit of the clamp loader of DNA polymerase III disrupts the interaction of primase with SSB [59,60,61,63], leading to the release of primase followed by the loading of the sliding clamp onto DNA by the clamp loader, and the binding of DNA polymerase III at the primer.

Of interest, small aromatic compounds named CFAM, BCBP, BOTP, and MPTA have been identified that interfere with bacterial growth by inhibiting the interaction of SSB with Exonuclease I, RecQ and PriA DNA helicase [27,28]. For MPTA, it acts as a structural mimetic of the Pro-Phe dipeptide in its C-terminal domain that interacts with the proteins mentioned above and with other replication proteins [28]. Other compounds more specifically inhibit the interaction of SSB with Exonuclease I compared with RecQ and PriA, but they too directly compete with Exonuclease I in binding to SSB [27]. These compounds also affect protein synthesis, suggesting that one or more of these interactions affects protein synthesis or that the compounds have other cellular effects.

## 5. DnaA

DnaA, DnaB, and DnaC act together to initiate DNA replication from the chromosomal origin (*oriC*) of *E. coli*. The role of DnaA is to recognize and assemble at sites in *oriC*, and then to unwind a region in *oriC* that serves as the entry site for DnaB helicase in a complex with DnaC (reviewed in [17,18,84,85,86]. A series of events then follow that lead to the establishment of the replication fork machinery that will duplicate the bacterial chromosome.

**Figure 1:**
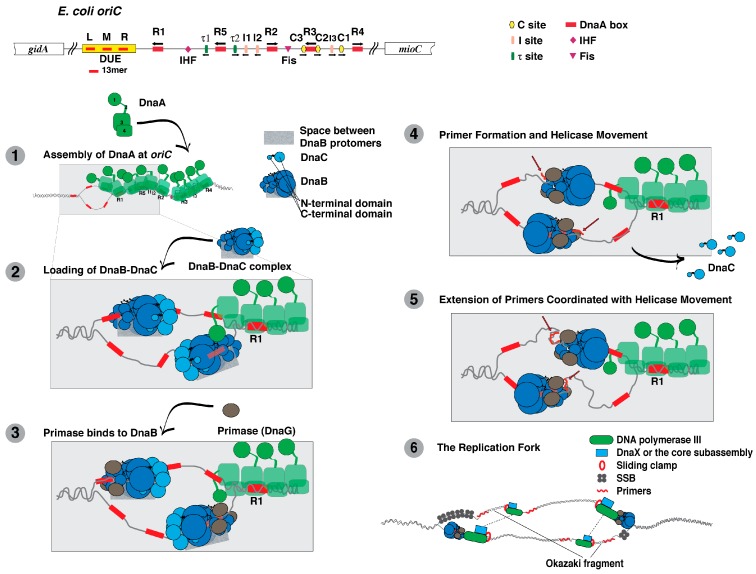
Replication initiation at the *E. coli* chromosomal origin involves the recruitment of DnaA, DnaB and DnaC to form the prepriming complex, followed by activation of DnaB, primer formation by primase, and DNA replication by DNA polymerase III holoenzyme. Shown at the top, the replication origin (*oriC*) of *E. coli* contains binding sites for Fis and IHF, and the DnaA boxes named R1-R5 that are recognized by DnaA in which the ATP or ADP bound to DnaA may affect the affinities to the respective sites [17,19,90,91]. In contrast, DnaA-ATP and not DnaA-ADP specifically binds to I-, τ- and C-sites. The sites named C3 and C2 overlap R3 and may be separate sites or part of R3 [17,19,90,91]. (**1**) DnaA, which has four functional domains as noted in the figure of DnaA, recognizes specific DNA sites in *E. coli oriC* to form a DnaA oligomer. DnaA then unwinds a region containing the 13mers named L, M and R; (**2**) Domain I of DnaA interacts with the N-terminal domain of DnaB in the DnaB-DnaC complex to load the complex onto the top and bottom DNA strands of the unwound region, forming a macromolecular entity named the prepriming complex. The shaded rectangle represents the space between adjacent DnaB protomers through which the single-stranded DNA passes during helicase loading; (**3**) Primase interacts with the N-terminal domain of DnaB, which is required for primer synthesis. In the transition to the next step, the open space between adjacent DnaB protomers presumably closes; (**4**) Primer synthesis (shown as red wavy lines) by primase on the top and bottom strands and the translocation of DnaB leads to the dissociation of DnaC from DnaB; (**5**) After primer synthesis, primase will dissociate from DnaB as the primer is bound by DNA polymerase III holoenzyme. DnaB will move to the junction of each replication fork; (**6**) DNA polymerase III holoenzyme extends the primers for the synthesis of each leading strand. DnaB at the junction of each replication fork unwinds the parental duplex DNA. The transient interaction of DnaB with primase as the helicase moves leads to the synthesis of subsequent primers that are extended by DNA polymerase III holoenzyme in the synthesis of Okazaki fragments. The dashed lines represent the contacts between two units of DNA polymerase III holoenzyme, forming a dimer in the coordinated synthesis of the leading and lagging strands.

Comparing its deduced amino acid sequence, DnaA is highly conserved among Gram-positive and Gram-negative bacteria [87]. On this basis, DnaA is thought to act similarly among bacteria. Biochemical studies reveal that DnaA is multifunctional. In addition to its binding to specific sequences at *oriC* named the DnaA box, τ, I and C sites, it binds to adenine-containing nucleotides, acidic phospholipids, and to other chromosomal sites (Figure 1). DnaA also interacts with a number of other proteins and self-oligomerizes. X-ray crystallographic analysis of a truncated DnaA (containing domains 3 and 4) of *Aquifex aeolicus* [88,89], and functional characterization of *E. coli* DnaA reveal that DnaA has four domains.

**Domain 1 (amino acids 1–90 of *E. coli* DnaA).** A number of proteins interact with Domain 1 of *E. coli* DnaA (reviewed in [18,92]), whose structure has been determined by NMR [93]. One is DnaB, which interacts with specific residues within this domain for its loading at *oriC* [93,94,95,96]. In addition, DnaA self-interacts via this domain in the process of DnaA oligomerization at *oriC* [93,97,98,99]. DnaA oligomer formation leads to the unwinding of *oriC* [90,98,99,100,101,102,103,104,105,106], and to the loading of the DnaB–DnaC complex onto this unwound DNA.

Domain 1 additionally interacts with the DNA binding proteins, DiaA and HU [107,108,109,110]. HU is major component of the bacterial nucleoid [111,112,113,114], whereas DiaA is a regulator of DnaA activity [108,115,116]. Both proteins stimulate the DnaA-dependent unwinding of *oriC* and DNA replication of plasmids containing *oriC* by stabilizing DnaA oligomerized at this site [108,110].

Ribosomal protein L2, which is an essential component of the ribosome, and Dps interact with domain 1 of DnaA [117,118]. Unlike the stimulatory effect of HU or DiaA on DnaA, L2 and Dps inhibit DnaA function. The L2 interaction interferes with the formation of the DnaA oligomer at *oriC* to inhibit the DnaA-dependent unwinding of *oriC*, and replication initiation in vitro [117]. Of interest, both ribosome biogenesis and DNA replication are highly coordinated with cell growth in *E. coli* and other organisms. These findings raise the possibility that, when the cellular abundance of L2 exceeds that needed for ribosome assembly, its inhibitory effect on DnaA reduces the frequency of initiation in order to coordinate ribosome biogenesis with DNA replication.

Dps protein is an iron-sequestering protein that is induced under conditions of redox stress or stationary phase growth [119,120]. Dps is thought to protect the chromosome of *E. coli* against iron- and hydrogen peroxide-induced free-radical damage. The interaction of Dps with DnaA that inhibits DnaA in replication initiation suggests that Dps acts as a checkpoint during oxidative stress to reduce initiations, providing an opportunity for mechanisms to repair oxidative DNA damage.

**Domain 2 (amino acids 90–130 of *E. coli* DnaA).** Among bacterial DnaAs, domain 2 varies in length and amino acid sequence. Apparently, this domain acts as a linker to join domain 1 with domain 3 [121]. In support, deletion analysis revealed that portions of domain can be removed without substantially affecting DnaA function [122,123,124]. However, DNA replication by respective mutant DnaAs in vivo appeared to be is less efficient than in wild type DnaA.

**Domain 3 (amino acids 130–347 of *E. coli* DnaA).** Domain 3 contains the Walker A and B boxes, and the sensor 1, 2 (box VIII) and box VII motifs shared by the AAA+ family of ATPases [125,126]. On the basis of the X-ray crystallographic structure of domains 3 and 4 of *Aquifex aeolicus* DnaA, domain 3 has two subdomains (reviewed in [127,128]). Domain IIIa resembles an abbreviated RecA-type fold joined to domain IIIb that consists of an antiparallel three-helix bundle. In contrast with other AAA+ ATPases, DnaA is a weak ATPase [129]. The crystal structure of domains 3 and 4 of *A. aeolicus* DnaA bound to the ATP analogue, AMP-PCP, reveals residues in the Walker A box that contact the β and γ phosphates of the bound nucleotide [89]. Like other ATP binding proteins, the crystal structure shows that specific Walker B box residues chelate magnesium ion complexed to ATP. Other biochemical and mutational findings support the role of the sensor 1, 2 and box VII motifs of DnaA in ATP binding, and ATP hydrolysis [130,131,132,133] (reviewed in [134]). Supported by biochemical studies, X-ray crystallographic data provide direct evidence of a conformational change; *A. aeolicus* DnaA complexed to ADP forms a toroid of six molecules, whereas the protein bound to AMP-PCP can be modeled as a right-handed helical filament [88,89].

Bis-indole derivatives that compete with ATP in binding to DnaA to inhibit its function in DNA replication of *oriC*-containing plasmids have been reported [108]. Of interest, the increased length of an aliphatic side chain of respective bis-indoles correlated directly with more effective inhibition, suggesting that these alkyl chains bind to a hydrophobic surface near the ATP binding pocket. Presumably, ATP analogues that bind to domain 3 of DnaA to inhibit DnaA function are likely to inhibit mammalian proteins that bind ATP. However, a BLAST search of UniprotKB using the sequence of domain 3 reveals that the closest human homolog is the ATPase NSF (N-ethylmaleimide sensitive factor), an AAA+ protein. Comparison of the cryo-EM structure of its ATP binding site (PDB 3J94) with that of DnaA shows that they are significantly different, with only four of the 23 residues within 5 Å of ATP sharing identity [135]. This very low similarity suggests the possibility of specific inhibitors of DnaA, an essential consideration if off-target side effects of novel antibiotics are to be avoided. 

**Domain 4 (amino acids 347–467 of *E. coli* DnaA)**. Binding of DnaA to the DnaA boxes in *oriC* and elsewhere in the chromosome is mediated by a basic loop followed by a helix-turn-helix motif in domain 4 [88,132,136,137,138,139]. Complemented by biochemical and mutational analysis of amino acids in this domain [137,138], X-ray crystallography and NMR analysis of domain 4 bound to the DnaA box sequence reveal specific residues that make contact with nucleotides of the DnaA box and with flanking nucleotides [88,139].

## 6. DnaB

DNA helicases have been organized into several superfamilies on the basis of shared amino acid sequence motifs [140,141,142]. DnaB is part of Superfamily 4 of DNA helicases that specifically function in bacterial and bacteriophage DNA replication [83,140,143,144]. By comparison, the eukaryotic replicative DNA helicase named MCM2-7 is a member of Superfamily 6. In the process of unwinding DNA, independent biochemical studies support the model that DnaB is bound at the replication fork to one of the two parental DNA strands [145,146,147]. Cryoelectron microscopy of *E. coli* DnaB [148,149], and X-ray crystallography of *Geobacillus kaustophilus*, *Geobacillus stearothermophilus* and *Bacillus subtilis* DnaB showed that the native structure of DnaB is a hexamer of identical subunits assembled as a toroid [81,150,151]. More recent studies revealed an alternate structure of an open right-handed spiral for *G. stearothermophilus* and *A. aeolicus* DnaB, but with constricted or dilated conformations of the N-terminal domain [146,152]. These different structures strongly suggest dynamic movement of its domains during DNA unwinding by DnaB. Each DnaB protomer has a larger C-terminal domain joined to an N-terminal domain via a linker α helix. The C-terminal domains of hexameric DnaB are nearest the junction between single-stranded and duplex DNA of an artificial replication fork [145,153,154].

Each C-terminal domain has a RecA-like fold that carries Walker A and B boxes and an arginine finger residue that bind and hydrolyze nucleotides to drive translocation and DNA unwinding [145,155,156,157,158]. Evidence indicates that DnaB and its homologues in other organisms translocate in the 5’-to-3’ direction on the single-stranded DNA to which they are bound [157,159,160,161]. Like other DNA helicases that are toroids [140,141,162,163,164], this DNA strand passes through the central cavity of DnaB, apparently interacting with specific residues that line the cavity during movement while the other parental DNA strand is excluded [146,147].

At *oriC* during the process of helicase loading, DnaB also interacts with DnaA and DnaC [18,93,94,95,96,165,166,167]. After helicase loading and translocation, DnaB bound to the lagging strand DNA template serves as a landing pad for primase followed by primer synthesis [77,168,169,170]. During replication fork movement, DnaB not only interacts with primase, but also with the τ subunit of DNA polymerase III holoenzyme to coordinate DNA unwinding with DNA synthesis by this DNA polymerase [171,172].

As with the vital roles of the replication proteins described herein, the essential function of DnaB in DNA replication makes it an attractive target for inhibition. The flavenol, myricetin, which is a natural plant product, has been described to inhibit the ATPase activity of *E. coli* DnaB by a non-competitive mechanism [30]. In support of this study, myricetin and similar flavenols were shown to impair the ssDNA-stimulated ATP hydrolysis by the closely related DnaB homologue of *Klebsiella pneumoniae* [173].

## 7. DnaC

Comparative genomics analyses place DnaA and DnaC in the replication initiator clade of proteins, and in the AAA+ family of ATPases [174,175] (reviewed in [176]). Its members bear several conserved amino acid sequence motifs named the Walker A and B boxes, and the sensor 1, 2 (box VIII) and box VII sequences that function in ATP binding and hydrolysis. Unlike DnaA that has a high affinity for ATP (K_D_~0.03 μM) [129], DnaC binds ATP weakly (K_D_~8 μM) [166,167,176]. Both DnaA and DnaC are weak ATPases. A unique feature of the initiator clade that also includes archaeal Orc1/Cdc6 and eukaryotic Orc2–5 is the initiator specific motif (ISM) comprising one or two helices located between the Walker A and B motifs of the AAA+ module [104,175,177,178,179,180]. In the case of DnaC complexed to ATP, the first α helix of the ISM is proposed to pack against the neighboring DnaC molecule in an oligomeric form of DnaC, causing the spiral assembly of DnaC protomers [177]. The role of the ISM is based on the X-ray crystallographic structure of the ATP binding domain of *A. aeolicus* DnaC, which has a similar arrangement in the helical filament model of domains 3 and 4 of DnaA in a complex with AMP-PCP [89]. 

DnaC does not act independently but must form a stable complex with DnaB (as the DnaB-DnaC complex) at the stage of replication initiation at *oriC*. Several independent studies strongly suggest that a site near the N-terminus of DnaC interacts with a specific surface in the C-terminal region of DnaB [181,182,183,184,185]. Formation of the DnaB-DnaC complex, which does not require a nucleoside di- or triphosphate bound to either protein, leads to as many as six DnaC molecules bound per DnaB hexamer [176,177,183]. As described above, DnaC monomers may interconnect to form a helical filament upon binding to DnaB, but other studies suggest that DnaC assembles as dimers onto DnaB [184,186]. On the basis that *A. aeolicus* DnaC also interacts with *A. aeolicus* DnaA, a separate model has been proposed that DnaC complexed to DnaB also interacts with DnaA oligomerized at *oriC* in the process of helicase loading [177].

Stimulated by ATP, DnaC is able to bind to single-stranded DNA, which is presumed to be required for its function in DNA replication [176,177,187,188,189,190]. Integrating these observations into the model described above, amino acid residues located in the inner channel of the DnaB-DnaC complex interact with the single-stranded DNA [177]. The interaction of DnaC with ssDNA may help to load the open-ring form of DnaB when complexed to DnaC onto the region of *oriC* unwound by DnaA [152]. Following the loading of the DnaB-DnaC complex at *oriC* to form a macromolecular complex containing DnaA, DnaB and DnaC, primer formation by primase leads to the dissociation of DnaC from DnaB and its activation as a DNA helicase [166,185,189].

## 8. Primase (DnaG)

In bacteria, primase (DnaG) synthesizes oligonucleotide primers (6–10 nucleotides) by recognizing preferred trinucleotide sequences in the lagging strand parental DNA as it emerges from DnaB while the helicase unwinds the parental duplex DNA (reviewed in [82,170,191]). In γ-proteobacteria, the preferred sequence is dCTG, in which the 5′ nucleotide of the primer corresponds with the central nucleotide of the template DNA [192,193]. Primase, which has three functional domains, relies on its ability to interact with the N-terminal domain of DnaB for primer formation (reviewed in [170]). Its C-terminal domain that is also called the helicase binding domain interacts with DnaB, whereas its RNA polymerase domain (RPD) containing a TOPRIM fold is responsible for primer synthesis [76,78,81,82,194]. Its zinc binding domain (ZBD) binds to DNA and is also proposed to recognize sites in the template DNA to initiate primer synthesis [195,196]. Of interest, the C-terminal domain of primase has a similar 3D structure as the N-terminal domain of DnaB but neither domain shares amino acid sequence homology [80,197,198].

As noted above, *G. stearothermophilus* DnaB is either a closed ring or an open spiral in which the interior channel formed by the N-terminal domains of each protomer is wider or dilated [81,146]. For the closed ring form of *A. aeolicus* DnaB, electron microscopic analysis revealed a narrow interior channel [199]. Primase appears to bind to DnaB in its dilated conformation in which the N-terminal domains of individual DnaB protomers are organized in a pairwise arrangement [81,146]. On the basis of FRET analysis together with crosslinking experiments and gel filtration assays, the ZBD of one primase molecule is able to interact with the RPD of a second primase molecule that is bound to DNA [194,200]. These observations support a model that two or perhaps three primase molecules bound to DnaB cooperate via their interactions with each other to select the site on the parental DNA strand followed by primer synthesis.

The interaction between primase and DnaB has a synergistic effect on their respective activities of primer synthesis and DNA unwinding [194]. In *E. coli*, this interaction is weak [80,201], suggesting a model in which primase interacts transiently with DnaB in the synthesis of primers. In hyperthermophilic bacteria, this interaction is stable as documented in studies characterizing the structure and activity of the DnaB-primase complex [194,197,202]. An extreme example is bacteriophage T7 gene 4 protein, which contains both primase and helicase activities in a single polypeptide that can then assemble into a homo-multimeric complex [203,204]. In summary, DnaB and primase coordinate their respective functions to unwind the parental DNA, and to lay down primers that are extended by DNA polymerase III in duplicating the chromosome.

On the basis of their very different chemical structures (Table 1), compounds discovered to inhibit *E. coli* primase apparently use unrelated mechanisms. For example, the phenolic monosaccharides extracted from the plant *Polygonum cuspidatum* is speculated to inhibit the binding of primase to ssDNA [32]. In comparison, the furans, imidazoles and pyrimidine derivatives were initially identified by in silico docking of compounds to the RPD of primase, followed by functional assays to demonstrate inhibition [33]. The mechanism of inhibition by the latter set of compounds has not been established.

## 9. DNA Polymerase I

The first DNA polymerase to have been discovered, DNA polymerase I not only polymerizes dNMPs from dNTPs by extending a primer end, it also has a proofreading exonuclease that removes misincorporated nucleotides, and a 5′-to-3′ exonuclease (reviewed in [205]). The activity of the 5′-to-3′ exonuclease together with its DNA polymerase activity is essential for its physiological role in the maturation of Okazaki fragments. Following the extension of RNA primers by DNA polymerase III holoenzyme in copying the lagging strand template of the parental DNA, the RNA of the resulting Okazaki fragment is removed by the 5′-to-3′ exonuclease of DNA polymerase I. This exonuclease is able to remove mononucleotides and oligonucleotides from the 5′-end of the Okazaki fragment. In concert with the removal of the RNA primer, DNA polymerase I extends the 3′-end of the upstream Okazaki fragment, resulting in the replacement of RNA with DNA. The abutting 3′- and 5′-ends after dissociation of DNA polymerase I are then joined together by DNA ligase.

## 10. DNA Polymerase III Holoenzyme

**Figure 2:**
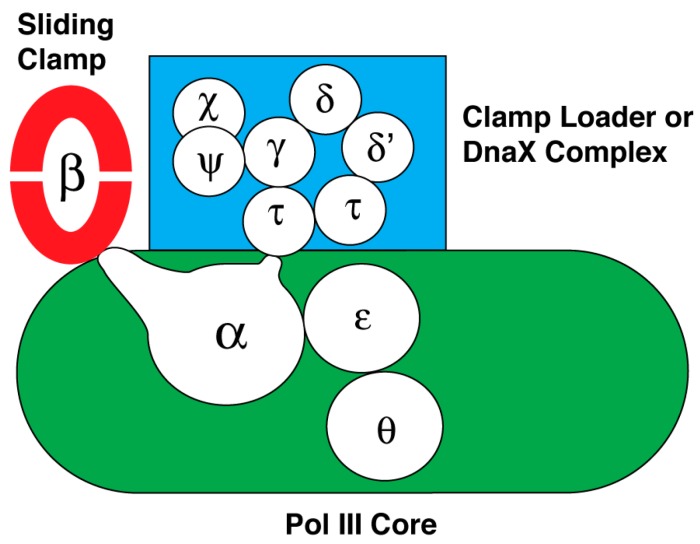
Subunit composition and their interactions of DNA polymerase III holoenzyme (reviewed in [207,218,224]). The subassemblies of DNA polymerase III holoenzyme are the sliding clamp composed of two DnaN or β subunits, the clamp loader or DnaX complex composed of seven subunits, and DNA polymerase III core containing the α, ε and θ subunits. The diagram also summarizes how these subunits interact within each subassembly and between subassemblies.

Like other organisms, *E. coli* has several DNA polymerases that have specific roles in DNA replication, DNA repair, or in extending DNA beyond DNA adducts that block DNA polymerase III holoenzyme [206,207]. Of these, DNA polymerase I and DNA polymerase III holoenzyme are essential for viability. As an overview of the latter, it is the cellular replicase composed of ten subunits that is responsible for duplicating the *E. coli* chromosome. Its subunits organize into three subassemblies named DNA polymerase III core, the sliding clamp, and the clamp loader or DnaX complex. The interactions between and among the subunits of this macromolecular machine are summarized in Figure 2. Believed to form a dimer in the replisome at the replication fork, it supports concurrent DNA replication of the leading and lagging strands [206,208]. However, recent studies indicate that the leading and lagging-strand DNA polymerases function independently [172].

*DNA polymerase III core.* The subassembly named core contains three subunits: α, ε and θ. The amino acid sequence of the α subunit encoded by the *dnaE* gene originally led to the placement of this DNA polymerase in a family of enzymes (Family C) that is separate and distinct from the other DNA polymerases of *E. coli* involved in DNA repair, and the DNA polymerases of eukaryotic cells (reviewed in [207]). More recent studies indicated that this subunit is a member of the X family of DNA polymerases that includes eukaryotic DNA polymerase β [209]. The α subunit also has the active site for DNA polymerization of dNMPs using dNTPs as substrates, and carries a domain named the clamp binding motif to be described in more detail below. This motif makes contact with a binding pocket in the sliding clamp to secure the DNA polymerase onto the DNA being copied so that the enzyme is highly processive [210] (reviewed in [207]). The α subunit also interacts with the single-stranded DNA template, the sliding clamp, and the τ subunit of the clamp loader as it synthesizes DNA [211,212,213,214]. Whereas this enzyme as a dimer is thought to synthesize both leading and lagging strands concurrently [206,208], Gram-positive bacteria with a low GC content uses two separate DNA replicases to copy the chromosome [49,215,216,217,218,219,220]. One named PolC is thought to synthesize both the leading strand and also the lagging strand. Its synthesis of the lagging strand follows after extension of RNA primers via the second DNA polymerase containing DnaE.

For the *E. coli* enzyme, the θ subunit has a role in its assembly with the α and ε subunits to form the core subassembly of *E. coli* DNA polymerase III [221,222,223]. This subunit is not universally found among bacterial species.

The 3′-to-5′ exonuclease of the ε subunit takes out an incorrectly inserted nucleotide after which the DNA polymerase continues DNA synthesis [225,226]. Unlike the core subassembly of *E. coli*, DNA polymerase III in which the polymerase active site is carried in a polypeptide that is separate from the ε subunit containing the proofreading exonuclease, the PolC DNA polymerase of Gram-positive bacteria contains the DNA polymerase, the proofreading exonuclease and the clamp-binding motif in the same polypeptide [217]. This proofreading activity is found in many DNA polymerases. 

*The sliding clamp*. The *dnaN* gene encodes the sliding clamp whose native form is a toroid containing two DnaN or β subunit protomers [227]. DNA passes through the central cavity of the toroid, tethering the core subassembly of DNA polymerase III on the parental DNA through the interaction between the clamp-binding motif in the α subunit of DNA polymerase III core and the binding pocket in a β subunit of the sliding clamp. This association confers to DNA polymerase core the ability to sustain DNA synthesis for several kilobases [228,229,230]. In contrast, the processivity of DNA polymerase III core without the sliding clamp is only about 10–20 nucleotides before it spontaneously dissociates from the DNA [231].

*The clamp loader*. The subassembly named the clamp loader, also known as the DnaX complex, is composed of δ, δ’, Ψ, χ, and three copies of DnaX protein [232,233,234]. Of these subunits, the δ, δ’, and DnaX subunits are AAA+ proteins [127,235,236]. Two versions of DnaX are either the full-length form (τ), or a truncated polypeptide (γ) that arises by ribosomal frameshifting at a specific codon that then leads to translational termination at a nearby stop codon [237,238,239]. Hence, the subunit composition of the clamp loader may be δδ’Ψχτ_3_, δδ’Ψχτ_2_γ, δδ’Ψχτγ_2_, or δδ’Ψχτγ_3_. Physiological evidence indicates that the native form of the clamp loader is δδ’Ψχτ_2_γ [206,208]. The function of the clamp loader is to load the sliding clamp onto DNA. A crystal structure of the clamp loader bound to a primed DNA template shows that δ,δ’, a truncated form of Ψ bound to one of the DnaX subunits, and three copies of DnaX protein (γ) form an open ring, which loads the sliding clamp onto DNA [240]. 

In a process that depends on the binding of ATP to the δ subunit of the clamp loader, the complex interacts with the sliding clamp via an interaction between the δ subunit of the clamp loader and the β dimer to load it onto DNA [217,232,233,234]. ATP hydrolysis by the δ subunit is coordinated with conformational changes in both the sliding clamp and clamp loader that lead to clamp loading. One collection of experiments suggests that the clamp loader pries open the β clamp by separating one of the interfaces of the β dimer [241,242,243,244]. A second set of studies suggests that the clamp loader traps the β clamp when the interface is transiently open so that the DNA can pass through [245]. The interface then closes.

Once the sliding clamp is assembled onto a primed DNA, it associates with the core subassembly of DNA polymerase III so that it can then extend the 3’-end of the primer to copy the parental DNA. Multiple interactions between and among proteins appear to be involved. An interaction between τ subunits of the clamp loader (δδ’Ψχτ_2_γ) and the α subunit dimerizes the core subassembly of DNA polymerase III for concurrent leading and lagging strand synthesis [246]; one core subassembly synthesizes the leading strand as the other synthesizes the lagging strand [206,208]. Two protomers of the τ subunit of the clamp loader also interact with DnaB helicase to enhance both the rate of nucleotide incorporation and the speed of unwinding by DnaB helicase [247,248,249]. In addition, the interaction between the χ subunit of the clamp loader and SSB stabilizes the DNA polymerase on DNA [59,60]. The proposed mechanism is described as a three-point switch. SSB bound to DNA interacts with primase, which is displaced from the primer it has synthesized by its interaction with the χ subunit of the clamp loader [61]. This displacement involves an interaction of the χ subunit with SSB.

The multiple interactions between subunits of DNA polymerase III holoenzyme and their binding with other replication proteins makes this enzyme an attractive candidate to develop new antibacterial compounds (reviewed in [20,21,22]). Inhibitory chemicals have been discovered, such as nucleotide analogs. Derivatives of 6-anilinouracil, benzyl guanine and 3-deazaguanine inhibit DNA polymerase III or both this enzyme and PolC of Gram-positive bacteria by interfering with the base pairing of dGTP with the cytosine base in the parental DNA to trap the polymerase bound to DNA in an inactive complex [48,49,50,51]. By comparison, BisQuinols (quinazolin-2-ylamino-quinazolin-4-ols) interfere with the binding of the enzyme to DNA [52]. In contrast, nargencin selectively inhibits DNA polymerase III of *E. coli* and *Staphylococcus aureus* [39]. Compounds (RU7-a thioxothiazolinine derivative [34], a biphenyloxime derivative [35], and nonsteroidal anti-inflamatory drugs [36]) that interact with the binding pocket of the sliding clamp at which a variety of proteins interact [250], and cyclic peptides that interfere with dimerization of the sliding clamp of *Staphylococcus aureus* have also been discovered [37]. More recently, griselimycin derived from *Streptomyces griseus* has been shown to inhibit the interaction between the sliding clamp and the α subunit of the enzyme of *Mycobacterium tuberculosis*, a serious multidrug-resistant pathogen [38]. The article by Pandey et al. in this series focuses on inhibitors of the sliding clamp of *H. pylori*.

Compared with these studies, which focus on DNA polymerase III or the sliding clamp, a high-throughput screen was performed that measured DNA replication in a biologically relevant system derived from *E. coli* [20]. It requires a single-stranded DNA bound by SSB, primer formation by primase that is dependent on recognition of a specific site in the single-stranded DNA derived from a bacteriophage, and extension of the primer by DNA polymerase III holoenzyme. Hence, the screen had the potential to identify inhibitors of all three proteins. In parallel, this assay was adapted to measure the activity of *B. subtilis* SSB, the sliding clamp and clamp loader activity provided by three subunits (τ, δ and δ’) of the DnaX complex, and PolC. For technical reasons, *B. subtilis* primase was omitted, but an oligonucleotide annealed to the single-stranded DNA served as the primer. The inhibitory chemicals were counter-screened against DNA polymerases of bacteriophage, *S. cerevisiae* DNA polymerase δ holoenzyme, and human mitochondrial DNA polymerase, leading to the identification of compounds that specifically inhibited either or both the *E. coli* DNA polymerase III and *B. subtilis* PolC, and also bacterial growth.

## 11. Topoisomerases of *E. coli*

Topoisomerases change the topological structure of DNA (reviewed in [251,252,253,254,255]). Their inhibitors are well known to impede the elongation phase of DNA replication. Specifically, quinolones lead to the formation of protein-DNA complexes that block replication fork movement [256]. An alternative mechanism of inhibition relies on the essential role of topoisomerases in regulating the superhelical density of the bacterial chromosome, which is affected by the process of DNA replication. Specifically, unwinding of the parental duplex DNA by DNA replication introduces positive turns elsewhere in the DNA. Topoisomerases are required to remove these topological constraints that would otherwise impede replication fork movement. Hence, compared with quinolones, other topoisomerase inhibitors in the aminocoumarin family indirectly interfere with DNA replication by acting as competitive inhibitors of ATP binding, presumably leading to an increase in positive superhelix density in the chromosome that inhibits fork movement. In addition, the topological state of *oriC* affects the initiation stage of DNA replication, but it is unclear whether topoisomerases are directly involved. Nevertheless, the intimate albeit apparently indirect connection of topoisomerases with DNA replication and the role of topoisomerase inhibitors as antibiotics merit discussion.

Topoisomerases are separated into two major groups. Type I topoisomerases introduce a transient break in one of two strands of duplex DNA, whereas type II enzymes create a transient break in both DNA strands. In *E. coli*, topoisomerase I and III are type I enzymes that are classified as type IA because they form an intermediate in which the enzyme is covalently bound to the 5′-end of DNA at the transient break. In contrast, type IB and IC enzymes form a covalent complex with the 3′-end of DNA. For the type I enzymes described here, the covalent complex is formed via an essential tyrosine residue, and ATP or its hydrolysis is not required for enzyme activity. Another major difference is that type IA enzymes serve as a bridge between the broken ends of DNA during strand passage, whereas type IB and IC enzymes act as a swivel.

In *E. coli*, topoisomerase III has been shown in vitro at a physiological salt concentration to be more effective than topoisomerase I and DNA gyrase at decatenation of interlocked circular DNAs [257,258,259,260]. The interlocked DNAs arise when positive supercoils that accumulate ahead of the replication fork redistribute on the circular DNA behind the forks, leading to catenanes when DNA replication is complete. A second pathway of catenane formation arises when replication forks converge near the end of DNA replication. Due to the interwound strands of duplex DNA of the unreplicated segment, the copying of this segment produces catenanes. Topoisomerase I has also been proposed to removed the negative supercoils that form behind RNA polymerase as it transcribes genes [261,262].

The type II enzymes of *E. coli* (specifically type IIA but cited below as type II for simplicity) are DNA gyrase and topoisomerase IV (reviewed in [254,255]). Together with topoisomerase I and III, these enzymes modulate the superhelical density of the bacterial chromosome [263]. Both are essential for viability, and bind and hydrolyze ATP as they change the topological structure of DNA. Of interest, DNA gyrase is unique among all topoisomerases in that it is able to introduce negative supercoils into DNA. The enzyme contains two GyrA subunits, wherein each subunit introduces a transient break in each strand of duplex DNA. A tyrosine residue in the active site of each GyrA subunit becomes covalently attached to the 5′-end of the broken DNA. Examination of the cleaved DNA reveals that the 5′ends have four nucleotides that are single-stranded, a characteristic of type IIA enzymes. Hence, DNA gyrase creates a staggered break in duplex DNA. The two GyrB subunits in the A_2_B_2_ tetramer bind and hydrolyze ATP; two ATP molecules are hydrolyzed in one cycle of DNA cleavage, passage of the intact duplex DNA segment through the transient break followed by rejoining of the broken DNA ends. ATP hydrolysis stimulates the transfer of the DNA through the transient break. It is thought that the physiological function of DNA gyrase is to remove the positive supercoils that would otherwise accumulate ahead of replication forks and transcription complexes. In addition, DNA gyrase may also facilitate the DnaA-dependent opening of *oriC* at the stage of replication initiation. The unwinding of *oriC* is required for the subsequent loading of DnaB helicase in a complex with DnaC.

Like DNA gyrase, topoisomerase IV is a type II enzyme [254,255]. Containing of two ParC and two ParE subunits, this enzyme was discovered by its role in the partitioning of daughter chromosomes to daughter cells prior to septum wall formation and cell division. Although the ParC and ParE subunits of topoisomerase IV are homologous to the GyrA and GyrB subunits of DNA gyrase, respectively, and is able to remove positive supercoils from DNA like DNA gyrase, it is unable to introduce negative supercoils into DNA. Its unique property is in the transfer of a duplex DNA through a transient break in another segment of DNA between two DNA molecules (intermolecular strand transfer). Hence, its physiological function is in the decatenation of linked daughter chromosomes that arise by DNA replication. In contrast, DNA gyrase by nature performs intramolecular strand transfer within a single DNA molecule.

Inhibitors have been discovered that act on DNA gyrase and topoisomerase IV (reviewed in [264]). Nalidixic acid and the aminocoumarins, novobiocin and coumermycin, are considered the first inhibitors found for these bacterial enzymes [265,266]. The successive generations of the quinolone family of antibiotics have led to the fluoroquinolones, which have improved cellular uptake mediated by membrane-bound porins. An example is ciprofloxacin that is widely prescribed to treat a broad spectrum of diseases caused by bacterial infection. These compounds inhibit DNA gyrase and topoisomerase IV by trapping the respective enzyme bound at the transient double stranded DNA break but prior to the step of rejoining the broken DNA ends, suggesting that fluoroquinones inhibit the subsequent step of DNA ligation. The covalent protein-DNA complex apparently blocks DNA replication forks, which leads to cell death [256,267,268]. Structures of the complex of moxifloxacin bound to topoisomerase IV of *Acinetobacter baumanii*, a Gram-negative pathogen, reveal the drug bound via a magnesium-water ion bridge with key residues of the GyrA subunit at the cleavage site, [269]. In comparison, the compound named GSK 299423 discovered by GlaxoSmithKline inhibits DNA gyrase of Gram-positive and Gram-negative bacteria by a mechanism that is distinct from the effect of fluoroquinolones [11]. It permits cleavage of one of two strands of duplex DNA, but blocks the subsequent step of double strand cleavage and the conformational change of the enzyme that follows. Related structures of quinoline pyrimidine trione-1 (QPT-1) bound to *Staphylococcus aureus* DNA gyrase reveal that the compound occupies the same site as moxifloxacin, but binds to residues of the TOPRIM domain of the GyrB subunit to inhibit religation [270]. Other novel compounds (quinolone NXL101 [271], NBTI5463 [272], pyrozole derivatives [273], and gyramides: *N*-benzyl-3-sulfonamidopyrrolidines [274]) have also been characterized. Derivatives of QPT-1 and GSK 299423 were in phase II clinical trials as of 2015.

The question of whether fluoroquinones target either DNA gyrase, topoisomerase IV, or both has been addressed genetically by selection for mutants resistant to a particular compound. Amino acid substitutions of Ser83 or Glu87 of GyrA, or the corresponding residues of ParC indicate that either subunit of the respective enzyme is the in vivo target [269,275]. Whereas inhibition of both enzymes decreases the likelihood of drug-resistant microbes arising by selection of mutations in both enzymes, such bacteria have emerged over the decades of fluoroquinone use.

The aminocoumarins (novobiocin, coumermycin and chlorbiocin) have been shown to interact with GyrB of DNA gyrase or ParE of topoisomerase IV (reviewed in [264,276,277,278]). These compounds bind to a site near the ATP binding pocket of these homologous proteins to compete with the binding of ATP. In contrast, simocyclinones bind to the GyrA subunit to interfere with the binding of DNA gyrase to DNA [279,280,281]. Unfortunately, the toxicity of these compounds precludes their clinical use.

## 12. DNA Ligase

*E. coli* has two DNA ligases. DNA ligase A is essential and functions in the joining of Okazaki fragments. DNA ligase B is dispensable for viability, but is suggested to play a role in the base excision repair pathway, or the mismatch repair pathway. Because DNA ligase A (and B) uses NAD^+^ as a cofactor and is phylogenetically distinct from the ATP-dependent eukaryotic DNA ligases, compounds that specifically inhibit DNA ligase A should avoid problems with toxicity in humans [282,283,284]. By high-throughput screening and by docking using the X-ray crystal structures of DNA ligase A, a variety of inhibitors (pyridochromanone, pyridopyrimidines, N-substituted tetracyclic indole, arylamino compounds and adenosine analogues) have been discovered that bind to a hydrophobic tunnel absent in human DNA ligases to compete with the binding of NAD^+^ [40,41,42,43,44,45,285]. More recently, the method of fragment-based drug design led to the identification of 6-azaindazoles as AMP-competitive inhibitors [286]. This method relies on the identification of low molecular weight compounds that bind to the target protein followed by determination of the binding mode of the molecule to the protein by X-ray crystallography or NMR spectroscopy, and chemical modifications to optimize binding [287]. Other substituted adenosine analogues have also been shown to inhibit DNA ligase A from diverse bacteria [46,47]. By comparison, arylamino compounds such as chloroquine used to treat malaria were found to selectively inhibit by a non-competitive mechanism, but their poor membrane permeability is suspected to hinder their use as antibiotics [45,288]. In other work, docking studies led to the identification of glycosyl ureides, glycosylamines, and tetracyclic indoles as inhibitors [42,43]. For the selected compounds tested, they act as competitive inhibitors for the binding of NAD^+^. In summary, DNA ligase A as a drug target is an active area where a major focus is on optimizing inhibitors that bind to its hydrophobic tunnel [284].

## 13. Conclusions

The structure of DNA led to the prediction that its duplication is via a semi-conservative process. In the decades of research that have passed since, we now know that the process in all domains of life is enzymatically mediated by macromolecular machines that act dynamically with the DNA as it is being copied. Because the individual proteins of bacteria of these nanomachines are substantially different from their eukaryotic counterparts, novel compounds that inhibit bacterial DNA replication are unlikely to affect DNA replication in eukaryotic cells, avoiding the problem of toxicity in humans [289]. Some bacterial proteins such as DnaA are highly conserved among all bacteria whereas others differ substantially, or for DnaC are only present in the *Enterobacteriaceae* family that includes *E. coli*. Hence, it is reasonable to expect that antibiotics will be found that are selective against a small group of related bacteria as well as other compounds that effectively inhibit diverse bacterial species. A high-throughput screen utilizing a reconstituted system that supports DNA replication from the *E. coli* replication origin potentially offers a very large number of targets that can be evaluated simultaneously and is very attractive (reviewed in [18]). The system also interrogates surfaces on individual proteins that must undergo a conformational change for DNA replication.

## Figures and Tables

**Table 1:** DNA replication proteins and their inhibitors.

*E. coli* Protein	Function	Inhibitor and Reference	
SSB	ssDNA binding	CFAM (2-[2-chloro-5-(trifluoromethyl)anilino]-5-methoxybenzoic acid) [27,28] BCBP (3-(tert-butyl)-1-(6-chloro-1,3-benzothiazol-2-yl)-4,5-dihydro-1H- pyrazol-5-one) [27,28] BOTP (2-[5-(3-bromobenzylidene)-4-oxo-2-thioxo-1,3,thiazoli- din-3-yl]-3-phenyl-propanoic acid) [27,28] MPTA ([5-(2-methyl-3-phenyl-2-propen-1-ylidene)-4-oxo-2-thioxo-1,3-thiazolidin-3-yl] (phenyl)acetic acid) [27,28]	
DnaA	recognition and binding to the *E. coli* replication origin (*oriC*)	bis-indoles (derivatives of 3-acetoxy-2,2′-bi-1H-indol) [29]	
DnaB	replicative DNA helicase	myricetin, a flavonol [30]	
Primase (DnaG)	primer synthesis	bicyclic 10-membered macrolide [31] phenolic monosaccharides [32] benzo[*d*]pyrimido[5,4-*b*]furans [33] benzo[*d*]imidazo[2,1-*b*]imidazoles [33] pyrido[3′,2′:4,5]thieno[3,2-*d*]pyrimidines [33]	
DNA polymerase I	removal of primers used for DNA synthesis		
DNA polymerase III holoenzyme, a DnaE-type DNA polymerase ^1^	DNA replicase	Subunit and Subassembly	Inhibitor and Reference
		DnaN or β subunit of the sliding clamp subassembly	RU7 [34], biphenyloxime [35], and nonsteroidal anti-inflamatory drugs [36]
		DnaN or β subunit of the sliding clamp subassembly of *S. aureus*	cyclic peptides [37]
		DnaN or β subunit of the sliding clamp subassembly of *M. tuberculosis*	griselimycins [38]
		α or DnaE subunit of the core subassembly of *E. coli* and *S. aureus* DNA polymerase III	nargencin [39]
DNA ligase A	ligation of Okazaki fragments	pyridochromanones [40] pyridopyrimidines [41] N-substituted tetracyclic indole [42,43] diamino-dimethylamino-pyrimido-pyrimidine [44] arylamino compounds (quinolones, quinacrines, bisquinolines) [45] adenosine analogues [46,47]	

^1^ DNA replicase containing PolC of Gram-positive bacteria are inhibited by 6-anilinouracil [48,49,50,51], BisQuinols [52], and benzyl guanine [53]. DNA polymerases of Gram-positive and Gram-negative bacteria bearing DnaE or PolC are inhibited by 3-deazaguanine [54].
